# A Three-Dimensional Phenotype Extraction Method Based on Point Cloud Segmentation for All-Period Cotton Multiple Organs

**DOI:** 10.3390/plants14111578

**Published:** 2025-05-22

**Authors:** Pengyu Chu, Bo Han, Qiang Guo, Yiping Wan, Jingjing Zhang

**Affiliations:** 1 College of Computer and Information Engineering, Xinjiang Agricultural University, Urumqi 830052, China; 2 Engineering Research Center of Intelligent Agriculture Ministry of Education, Urumqi 830052, China; 3 Xinjiang Agricultural Informatization Engineering Technology Research Center, Urumqi 830052, China

**Keywords:** cotton, plant phenotype, artificial intelligence, residual module, point cloud segmentation

## Abstract

Phenotypic data of cotton can accurately reflect the physiological status of plants and their adaptability to environmental conditions, playing a significant role in the screening of germplasm resources and genetic improvement. Therefore, this study proposes a cotton phenotypic data extraction algorithm that integrates ResDGCNN with an improved region-growing method and constructs a 3D point cloud dataset of cotton covering the entire growth period under real growth conditions. To address the challenge of significant structural variations in cotton organs across different growth stages, we designed an innovative point cloud segmentation algorithm, ResDGCNN, which integrates residual learning with dynamic graph convolution to enhance organ segmentation performance throughout all developmental stages. In addition, to address the challenge of accurately segmenting overlapping regions between different cotton organs, we introduced an optimization strategy that combines point distance mapping with curvature-based normal vectors and developed an improved region-growing algorithm to achieve fine segmentation of multiple cotton organs, including leaves, stems, and flower buds. Experimental data show that, in the task of organ segmentation throughout the entire cotton growth cycle, the ResDGCNN model achieved a segmentation accuracy of 67.55%, with a 4.86% improvement in mIoU compared to the baseline model. In the fine-grained segmentation of overlapping leaves, the model achieved an R^2^ of 0.962 and an RMSE of 2.0. The average relative error in stem length estimation was 0.973, providing a reliable solution for acquiring 3D phenotypic data of cotton.

## 1. Introduction

Cotton is an important crop that provides valuable raw materials for the global textile industry [1]. Research on cotton phenotypes enables a more accurate understanding of the physiological status of plants, their adaptability, and responses to environmental changes [2], which is critical for breeding selection and production. The application of advanced technologies, such as deep learning, in plant phenotyping plays a significant role in improving cotton yield and overall agricultural productivity [3].

Plant phenotyping refers to the comprehensive quantitative assessment of complex traits, such as development, growth, resistance, tolerance, physiology, structure, yield, and ecology [4]. A variety of methods are available for phenotype extraction. Traditional approaches primarily rely on manual measurements, which are often destructive to plant structures, time-consuming, labor-intensive, inefficient, and difficult to scale up, thus limiting their accuracy [5]. In recent years, phenotyping research has primarily focused on two-dimensional data. With the advancement of digital image processing technologies, visible light imaging has been widely used in plant phenotypic analysis. Due to its low equipment requirements and ease of operation, 2D image-based phenotyping methods have found broad applications in agricultural engineering. However, despite their success, 2D-based phenotyping approaches face many limitations. Due to the complex structure of most plants, which involves issues such as shading, overlapping, and multiple branches, it is difficult to obtain accurate parameter information using only two-dimensional images. In contrast, 3D point clouds contain depth information that complements the limitations of 2D images, largely overcoming the challenges of inaccurate measurements caused by single-view imaging. To address the aforementioned limitations, 3D imaging systems for high-throughput plant phenotyping have garnered increasing attention from plant researchers in recent years [6]. In this context, the acquisition and analysis of 3D structural phenotypic information for cotton plants has emerged as a research hotspot.

A prerequisite for effective phenotypic trait extraction is the availability of reliable and efficient segmentation methods. With the advancement of high-performance hardware and neural network architectures, 3D deep learning-based approaches have demonstrated significant potential in improving the generalizability and accuracy of part segmentation in recent years [7]. Previous studies have achieved promising results in the segmentation of crop point clouds at static or specific growth stages. For instance, Deng et al. [8] proposed a precise stem segmentation method for pumpkin seedlings and achieved satisfactory results on a point cloud dataset constructed from collected pumpkin seedlings. Shen et al. [9] realized accurate segmentation of cotton phenotypic organs at the seedling stage, while Zhang et al. [10] implemented automated point cloud segmentation of cucumber seedlings from individual plants down to the organ level. Moreover, Yan et al. [11] successfully achieved efficient and accurate point cloud segmentation of stem–leaf structures in tomato, soybean, and cotton. However, cotton plants contain six distinct organs—leaves, stems, main stems, flower buds, flowers, and bolls—throughout their full-growth cycle. Due to the substantial morphological variations across different developmental stages, existing segmentation methods still face challenges in achieving robust adaptability over the entire growth period.

In addition to plant organ segmentation, precise segmentation of individual organs plays a pivotal role in phenotypic trait extraction, as its accuracy directly affects the reliability of the extracted traits. Liu et al. [12] reconstructed 3D point clouds of rapeseed plants using a multi-view stereo (MVS) approach and extended existing Euclidean distance and spectral clustering algorithms. They also employed an iterative method to achieve precise segmentation of individual organs. Lin et al. [13] proposed a spatial clustering segmentation method for field crop rows, enabling complete extraction and segmentation of individual plants from crop population point clouds, thus facilitating automated measurement of individual crop phenotypic parameters. Peng et al. [14] utilized a Laplacian contraction algorithm to reconstruct plant skeletons from single-plant tomato point clouds obtained by robotic systems. After skeleton correction, the structure was decomposed into sub-skeletons representing stems and leaflets, enabling precise segmentation between stems and petioles. Additionally, a region-growing-based mean shift clustering method was applied to distinguish between leaves and petioles. While existing methods exhibit limited adaptability when applied to cotton, a crop characterized by highly complex structures and intertwined branches and leaves. Therefore, there is an urgent need for a high-precision and efficient algorithm tailored for precise segmentation of individual organs across the entire growth cycle of cotton, aiming to improve both segmentation accuracy and general applicability.

The extraction of plant phenotypes is an important criterion for evaluating the accuracy of model reconstruction and segmentation [15]. Parameters such as leaf length [16], leaf width, area [17], and stem length [18] are key indicators for monitoring plant growth and predicting yield. In recent years, significant progress has been made in cotton phenotypic extraction, and it has been widely applied in the analysis of cotton morphological characteristics, such as the automatic assessment of plant height and leaf area. However, studies focusing on the bell drop rate—a critical phenotypic trait—are still relatively scarce. Some studies have analyzed bud abscission through manual labeling and traditional image recognition techniques, but these methods often rely heavily on manual intervention and have low processing efficiency in practical applications. How to accurately extract the number of buds and track their dynamic changes using 3D technology and point cloud data remains a major challenge at present.

Currently, full-period cotton phenotypic extraction faces three major challenges. (1) Cross-period generalization capability: The morphology of cotton organs varies significantly across different growth periods, involving leaves, stems, flowers, and bolls. Existing methods lack sufficient generalization ability for full-period segmentation, making it difficult to accurately extract each organ. (2) Self-occlusion in 3D space: Severe occlusion of cotton leaves hinders the precise segmentation of small organs, thereby affecting the accuracy of leaf and bud growth monitoring and bell drop rate estimation. (3) Insufficient adaptability for dynamic extraction: Most existing phenotypic extraction methods target a single growth period and perform poorly across the entire growth cycle, making it difficult to accurately capture the dynamic changes of key phenotypic parameters such as plant height and stem length, which limits their application in precision agriculture.

To address the above issues, this study conducts an in-depth investigation and proposes the following solutions:

1. A novel full-period cotton organ segmentation model is proposed. The network architecture is optimized based on DGCNN and integrated with residual modules to enhance feature extraction capabilities. This method significantly improves the segmentation accuracy of cotton organs across all growth periods, achieving a 4.86% increase in mIoU compared to baseline models.
2. An improved algorithm for precise segmentation of individual organs based on region growing is developed. By integrating point-to-point distance mapping and curvature-normal features, the method effectively addresses the problem of organ overlap in cotton, enabling precise segmentation of organs, such as leaves, stems, and buds. In the most challenging task of overlapping leaf segmentation, the method achieves an R^2^ of 0.962 and an RMSE of 2.0. Based on this improved algorithm, the bell drop rate is innovatively calculated, providing a novel technical approach for cotton growth monitoring and yield estimation.
3. A phenotypic computation framework applicable to different growth periods is developed. By calculating plant height and stem length and comparing them with ground-truth measurements, the framework achieves a mean relative error of only 0.973, fully demonstrating its effectiveness in extracting key phenotypic parameters. This provides reliable support for precision agriculture and intelligent breeding.

## 2. Materials and Methods

### 2.1. Experimental Materials and Data Collection

**Figure 1:**
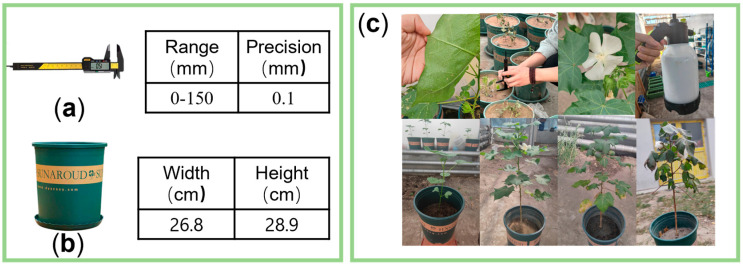
Cotton growing greenhouse and data collection parameters. (**a**) Accuracy gauge. (**b**) Flowerpot. (**c**) Cotton greenhouse growing.

The h33-1-4 cotton variety was selected for cotton planting and point cloud data collection. Seeds were planted in six pots, with 3–4 seeds per pot at 3–4 cm above the soil level. These pots were then placed in a greenhouse where the temperature was maintained between 25 °C and 27 °C. The seeds were planted in six pots, with 3–4 seeds per pot at 3–4 cm above the soil level. For each pot, after the germination of seeds, one well-grown cotton plant in each pot was selected from the seedling stage (young leaves emerged and spread out), watered at ten-day intervals, fertilized and dosed during the bud stage. Planting was ended at the spitting stage. From 5 May 2024 (emergence stage) to 22 October 2024 (flocculation stage), data collection was carried out at 3–4 day intervals in the greenhouse, as shown in Figure 1a.

Specifically, a smartphone was used as the acquisition device to capture images of cotton plants. Videos were recorded with a total duration of approximately 40 s and around 1200 frames, including one full rotation each from top-down, horizontal, and bottom-up perspectives. To prevent motion blur, the camera was moved slowly during filming. An appropriate distance was maintained to ensure that the entire cotton plant remained within the frame in each shot, thereby providing more comprehensive information on object shape and surface reflectance. Plants were selected under favorable lighting conditions and with distinguishable textures to achieve better reconstruction results. The device was specifically configured with the following settings: resolution set to 4000, gridlines and leveling assistant enabled for shooting, video stabilization turned off, and the “HDR video” option disabled.

Cotton phenological parameters were measured manually after shooting using digital simple calipers, as shown in Figure 1a. In order to ensure cotton development without disrupting the growth of cotton, plant height (vertical height from the flowerpot as a horizontal line to the highest point of the plant), and the length of the main diameter (the main diameter breaks out of the soil to the bifurcation of the terminal leaf stalks) were measured in a total of 20 sets of data to evaluate the segmentation performance of the segmentation network on the cotton dataset. Plant phenotypic parameters were calculated using the flower pot as a reference, as shown in Figure 1b.

### 2.2. Data Composition

#### 2.2.1. Point Cloud Data Acquisition

**Figure 2:**
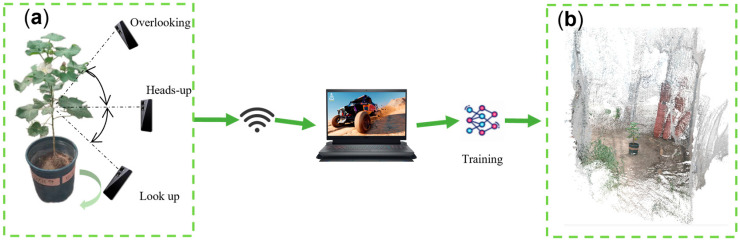
Data acquisition and nerf reconstruction. (**a**) Data acquisition. (**b**) Reconstruction results.

**Figure 3:**
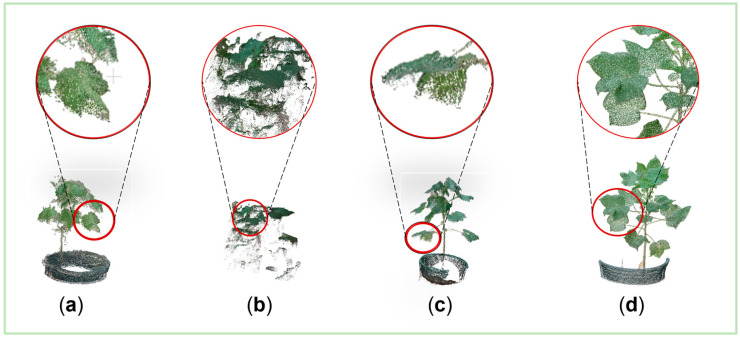
Cotton point cloud rekeying results. (**a**) Limited lighting conditions. (**b**) Motion blur. (**c**) Environmental effects. (**d**) Ideal conditions.

The accuracy and effectiveness of 3D reconstruction [19] directly affect the extraction of plant phenotypes and analysis of traits. High-precision 3D reconstruction can more realistically capture the details of cotton, such as the morphological structure of stalks, leaves, buds, and cotton peaches, and provide better cotton segmentation data. Three-dimensional reconstruction was performed using Luma AI, a platform based on NERF technology [20]. The data grown in the greenhouse are captured by a smartphone and the captured video is fed into the platform, which synthesizes a continuous 3D scene using deep learning by modeling the complete volumetric radiation field [21], and finally uses neural network’s point-of-view rederivation [22] to form a realistic scene as shown in Figure 2b. The reconstruction process is as follows:

The results of the reconstruction can be seen on the left side, with the problems encountered by the reconstruction. That is, the point cloud files are generated due to lighting conditions, motion blur, and environmental effects conditions, as shown in Figure 3a–d. On the right side is the point cloud file generated after the improvement. The model state is complete but the local point cloud distribution is scattered with many noise points, and the details of the radial lobe and other organs are not obvious. Figure 3b contains only the top foliage information, with no organs, such as stems and leaves, below the top, and the main body is missing. The leaf surface is incomplete, with insufficient texture details. Figure 3a was influenced by light conditions and reconstructed from data collected in the greenhouse at dusk. In this condition, the lighting conditions blur the plant texture details and affect page layering reconstruction. Figure 3b is affected by motion blur, where data collection was conducted at a high speed, causing distortion of the image, resulting in missing plant phenotypic details. Figure 3c shows the effects of the environment, with the data collected under wind speed interference conditions. Cotton foliage is shaken by wind speed, and the plant morphology transforms as a result. Morphological transformations affect the integrity of the reconstructed organ.

In general, the cotton plants were captured using the acquisition equipment. The data should be captured at a slow and steady rate under good light conditions and under stationary plant conditions from multiple viewpoints, as in Figure 3d, multiple angles, and maintaining a suitable distance. As shown in Figure 3d, it is important to be in a room with good light conditions, no wind and no interference. The video was taken from a top view, flat view, and elevation view in one turn each. The shooting process moves slowly; the single plant shooting time is 40 s. The appropriate shooting distance of the reconstruction results of the collected data should be maintained. The reconstructed cotton plant is rich in details, the object shape is complete, the texture is clear and the rendering effect is good.

#### 2.2.2. Data Preprocessing

High-quality data construction is crucial for the training of deep learning models. Although the 3D point cloud generated by NERF technology has high fidelity [23], there is a large amount of redundant information and environmental noise at the same time, which affects the effective learning of target features by the model. Therefore, it is important to establish an efficient data processing process to improve the accuracy and efficiency of cotton phenotyping research.

**Figure 4:**
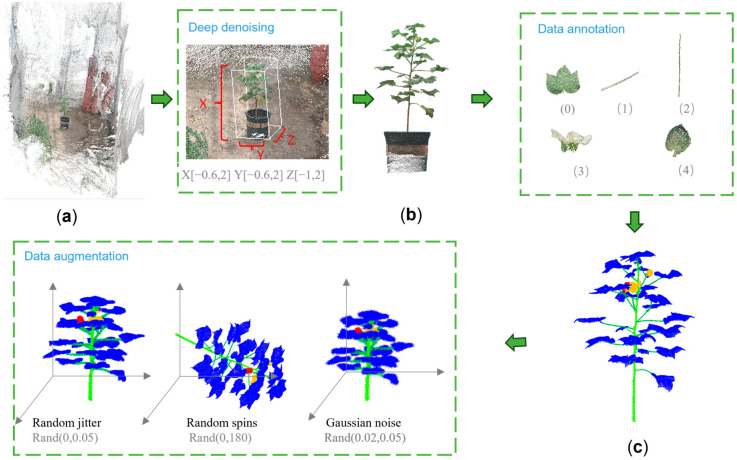
Cotton point cloud data composition process. (**a**) Reconstruction diagram. (**b**) After marking. (**c**) After annotation. (0–4) Labeled values for each organ.

In this study, four steps were used for data construction and optimization: point cloud denoising, data classification, data annotation and data enhancement, as shown in Figure 4.

(1) Point cloud denoising

The point cloud data generated by NERF reconstruction inevitably contain noise points (outliers and background points), which seriously interfere with the performance of the leaf segmentation network, as shown in Figure 4a. Traditional point cloud denoising methods, such as conditional filtering, statistical filtering and voxel filtering relied on by FARO 3D LiDAR scanning [24] have low processing efficiency and are not applicable to the point cloud data characteristics generated by NERF. To address the above problems, this study proposes an efficient denoising method based on the depth segmentation of coordinate axes based on the implicit representation properties of the volume field of NERF, as shown in Figure 4 (deep denoising). Specifically, the cotton plant point cloud model is regarded as the center object, a 3D coordinate system is established, and the range of values of the XYZ axes is limited, so as to eliminate the background noise in the spatial dimension and retain only the main body of the cotton plant (including the flower pot), as shown in Figure 4b. This method significantly improves the accuracy of the subsequent segmentation task while preserving the structural integrity.

The depth filter function is defined as follows:

(1) $$D_{\left\{filtered\right\}}=\left\{P\in P_{cloud}|D_{\left\{min\right\}}\leq D\left(P\right)\leq D_{\left\{max\right\}}\right\}$$

In Formula (1), $P_{cloud}$ denotes the original point cloud dataset. P denotes a point in the point cloud. $D\left(P\right)$ denotes the depth value of the point P. $D_{\left\{min\right\}}$$D_{\left\{max\right\}}$ are the minimum and maximum thresholds for depth filtering, respectively. $D_{\left\{filtered\right\}}$ is the point cloud dataset after depth filtering.

(2) Data classification

**Figure 5:**
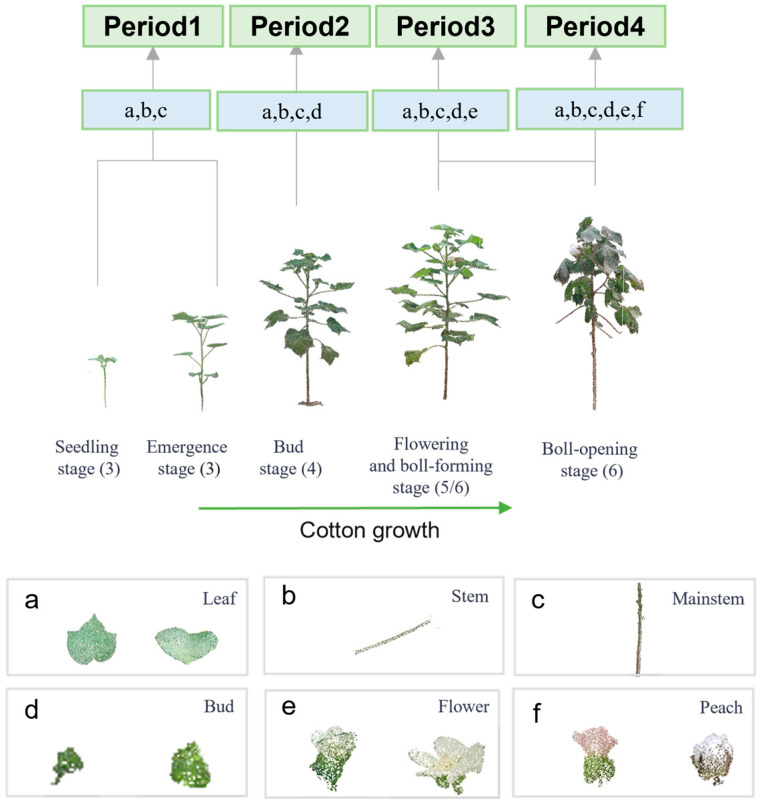
Cotton period classification.

In order to achieve accurate segmentation of cotton organs throughout the reproductive cycle, it is necessary to ensure that the data have a consistent number of labels in different periods. Cotton organs include six categories: leaves, stems, main stems, buds, flowers and bolls, as shown in Figure 5a–f. There are significant differences in the morphology, structure and size of organs at different stages of growth, so it is necessary to rationally stage cotton samples based on the number of organ types.

According to the five main fertility stages of cotton—seedling, seedling, bud, boll stage, and flocculent stages—the maximum number of organ types were 3, 3, 4, 6, and 6, respectively. Considering that the organ types of the boll stage and flocculent stage overlap, and the number of organs has overlapping stages, the whole fertility period was divided into four periods in this study.

Period 1 contains the emergence and seedling stage, with a total of 3 types of organs; period 2 corresponds to the bud stage, containing 4 types of organs; period 3 is the early boll stage (before bolls are formed), containing 5 types of organs, as shown in Figure 5e; and period 4 is the late boll stage and fluffing, when the bolls are already present, with a total of 6 types of organs, as shown in Figure 5f.

Based on the emergence of organs for the manual labeling stage, the resulting period labels are used both for the training and validation of the period classification model and as the data construction basis for the organ segmentation model to ensure the generalizability and robustness of the model under different growth stages.

(3) Data labeling

The preprocessed cotton point cloud data lacked sufficient segmentation information and, therefore, could not be directly input into the neural network for training. To solve this problem, the preprocessed point cloud data were labeled by assigning appropriate labels to each point cloud sample based on the different shape characteristics of the plant phenotypes, thus creating a dataset that meets the training requirements, as shown in Figure 4c. The point cloud data of cotton were segmented and labeled using CloudCompare v2.13 beta software. The labeling was performed according to the cotton organs, i.e., leaves (0), diameter (1), main diameter (2), buds (3), and bolls (4); in total, five categories were assigned labels, as shown in Figure 5. From the segmented point cloud data, 300 point cloud samples of cotton plants were selected and divided into training and test sets with a data ratio of 4:1.

(4) Data enhancement

**Figure 6:**
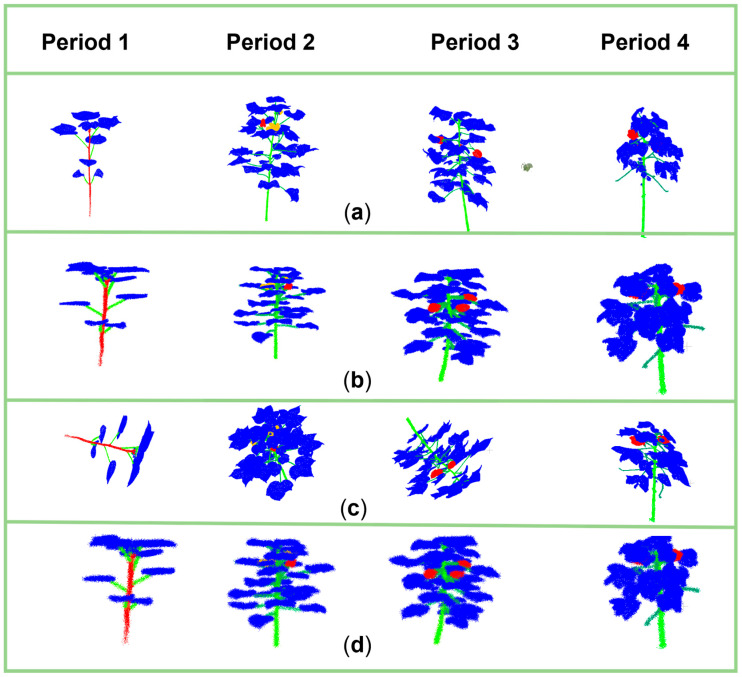
Cotton point cloud data results. (**a**) Point cloud annotation. (**b**) Random jitter. (**c**) Random Rotation. (**d**) Gaussian Noise.

In order to improve the generalization ability of the model and enhance its adaptability in complex environments [25], this study performed three data enhancement operations: random jitter, random rotation and Gaussian noise on the training set data that had been divided, as shown in Figure 6a–d. (1) In Gaussian noise addition, by introducing random noise into the point cloud data, the perturbation amplitude is controlled between [0.02, 0.05] meters, which simulates the measurement errors that may occur in the actual acquisition process, and helps to improve the model’s fault tolerance in the noise environment. (2) In random rotation, taking the geometric center of the cotton point cloud plant as the axis, the point cloud is randomly rotated around the *Z*-axis, and the rotation angle is sampled uniformly in the [0°, 180°] interval. This method effectively extends the spatial distribution range of the data and improves the model’s ability to recognize plants with different orientations. (3) In random dithering, small-amplitude perturbations are added to the 3D coordinates of each point, with the perturbation amplitude controlled between [0, 0.05] m, which further simulates the sensor acquisition error and enhances the robustness of the model to local changes.

With the above enhancement strategy, the generated training samples are closer to the point cloud distribution in real scenarios, significantly improving the training stability of the neural network model, as shown in Figure 6.

### 2.3. Point Cloud Segmentation

#### 2.3.1. Cotton Point Cloud Segmentation Process

**Figure 7:**
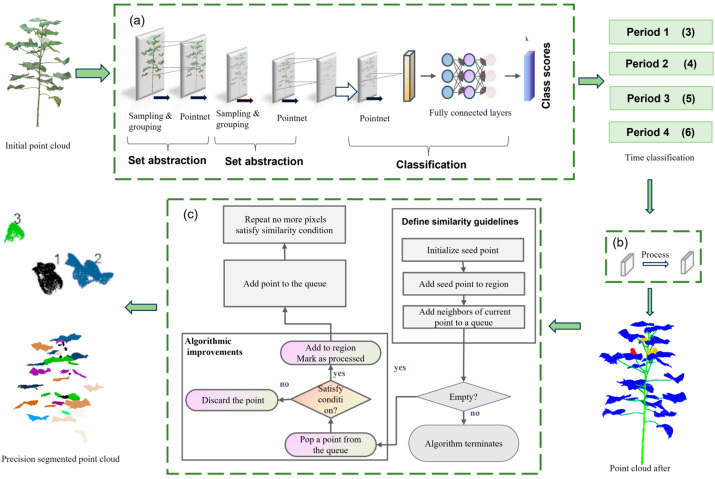
Cotton point cloud segmentation process. (**a**) Staging of cotton. (**b**) Organ segmentation. (**c**) Precise segmentation of individual organs.

The extraction of phenotypic traits in cotton needs to be based on organ-level structural analysis, i.e., the extraction of relevant features from the smallest units (Single leaf, single boll). To achieve this goal, plant data in the form of point clouds must be subjected to refined organ-level segmentation [26]. The overall segmentation process is divided into three stages: period classification, organ segmentation and precise segmentation of individual organs. The specific steps are shown in Figure 7. Firstly, the original cotton point cloud data are classified [27] into periods using PointNet++. The PointNet++ [28] network implements multi-scale sampling, grouping and feature extraction through the Set Abstraction module to capture local geometric structures at different scales; subsequently, the network utilizes a PointNet [29] module to extract global features, and through the fully-connected layer, outputs the period categories. The categorization results classify the data into four growth periods, laying the foundation for subsequent organ segmentation. After the period classification is completed and labeled, the organ segmentation of the point cloud data is performed using the ResDGCNN model. ResDGCNN constructs a dynamic graph convolution structure [30] to mine the local spatial relationships in the point cloud to achieve a fine distinction between the organ categories of leaves, stems, main stems, flower buds, bolls, etc. Finally, for the segmented regions of similar organs, the improved region growth algorithm is further used to realize precise segmentation of individual organs, i.e., differentiating and numbering similar organs (multiple leaves, multiple cotton bolls) at different locations. Finally, through the statistical and visual analysis of the segmentation results, the segmentation effect of the model and the accuracy of organ recognition are evaluated intuitively.

#### 2.3.2. Cotton Organ Segmentation Architecture

**Figure 8:**
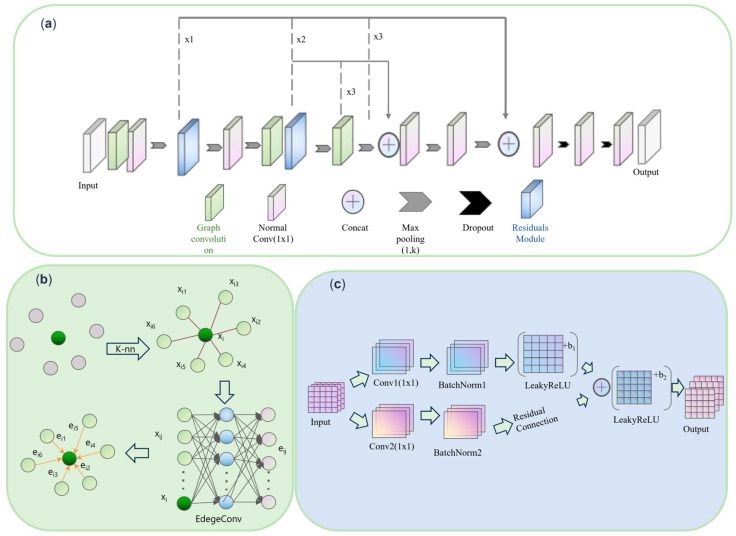
Cotton organ segmentation architecture. (**a**) Overall network architecture. (**b**) Graph Convolution Module. (**c**) Residual Module.

The original DGCNN model fails to fully consider the differences in organ morphology in different periods in dealing with the point cloud segmentation task for the full cotton reproductive cycle, resulting in its unsatisfactory performance on the full cotton data. In order to improve the model’s segmentation accuracy of the organs at each growth stage of cotton, this paper improves the DGCNN network architecture and proposes a ResDGCNN network for the full-growth period of cotton, whose overall structure is shown in Figure 8a.

The improved network takes the sampled cotton point cloud containing spatial location information as input, which are sequentially processed by the graph convolution module, convolution layer, residual block [31], and feature fusion module. First, in the graph convolution module, the spatial structure relationship between points is introduced by constructing a local neighborhood map, which effectively enhances the expression of local context information. Subsequently, feature information is further extracted and refined in the ordinary convolutional layer to enhance the model’s ability to perceive local geometric details. In order to alleviate the problem of gradient vanishing and feature degradation that may be brought by the deep network, the network introduces the residual structure. The residual module retains the original input information through constant mapping, effectively reduces the loss of features in the multi-layer transfer, and enhances the depth of information expression of the network. The residual module in the second stage learns high-level semantic features while continuing to retain the underlying geometric features to achieve the co-optimization of deep and shallow information.

After the feature extraction stage is completed, the feature fusion module integrates the features from different levels to improve the model’s adaptability to multi-scale structures and the comprehensive understanding of complex point cloud scenarios. Thanks to the above design, ResDGCNN is able to more accurately recognize and segment cotton organs with different growth periods and morphologies and shows better segmentation performance in the full-growth cycle of organ segmentation of cotton.

(1) Graph Convolution Module

Graph Convolution Networks (GCNs) significantly improve the geometric information representation of point cloud data by modeling the interrelationships between points and effectively extracting both local and global features, as shown in Figure 8b. Taking the feature vector of each point as input, the normal vector, through the spatial coordinates of the points, provides the base input for graph convolution, enabling the network to perform further processing and learning based on these features. The k-NN algorithm [32] (K-Nearest Neighbor) is used to find the k nearest neighbors of each point i. The feature vector of the center point is set to be *x_i_*, and the distance to the neighboring point *j* is denoted as $x_{ij}$. The distance between each point and the other points is calculated to build the adjacency matrix. In the next step, EdgeConv is performed to update the feature representation of the points by calculating the “edge features” between each point and its neighbors. Edge features capture the relative changes between a point and its neighbors and can effectively improve the perception of local structures in the point cloud. The formula for edge convolution is as follows:

(2) $$e_{ij}=\phi \left(\theta \cdot \left(x_{i}-x_{ij}\right)\right)+\psi \left(\theta^{\prime }\cdot x_{ij}\right)$$

In Formula (2), $e_{ij}$ denotes the elements of the feature map matrix $E_{ij}$, $x_{i}$ is the eigenvector of center point i, and $x_{ij}$ is the eigenvector or distance of point j with respect to point i. ϕ and ψ are the activation functions, while θ and $\theta^{\prime }$ are the convolution kernel parameters used to compute the feature maps between points and their neighbors.

The point cloud after edge convolution newly contains the correlation information between the point clouds. The discrete and disordered point cloud data are transformed into a graph-like structure, which ensures that each point in the point cloud contributes to the network independently and disordered. It enables the network to efficiently capture the local geometric features in the point cloud and improves the network’s ability to perceive the local shape and structure of the point cloud.

(2) Residual Convolution Module

In order to improve the performance of the network in processing complex features, the residual convolution module is introduced in this paper, as shown in Figure 8c. The input features are processed through two paths aiming to enhance the diversity and nonlinearity of feature extraction, thus improving the feature representation of point cloud data. One path processes the input features through 1 × 1 convolution, batch normalization and the LeakyReLU activation function to output enhanced features. This process adjusts the number of channels and avoids overfitting while maintaining training stability. The other path performs only batch normalization after convolution to further preserve the spatial structure of the features and enhance the network’s ability to capture local features. The synergy of the two paths enables the network to extract features from different levels and scales, which enhances the accuracy and diversity of the feature representation. The output features of the two paths are fused through residual connections. This helps alleviate the problem of gradient vanishing in deep networks and improves the flow of gradients during backpropagation. The residual connection allows the network to converge faster during training, improving training efficiency and avoiding information loss. Finally, after one LeakyReLU activation function processing, the output features are nonlinearly transformed to further enhance the feature expression ability. In point cloud learning, the residual module significantly improves the learning performance of the network by introducing nonlinear feature mapping and enhanced feature fusion, enabling the model to handle deeper features. The residual convolution module in the global network is better able to capture both local and global information in the data, thus enhancing the learning ability and performance of the network.

#### 2.3.3. Improvement of the Region Growth Algorithm

After completing the organ-level segmentation, the cotton point cloud data still need to be further processed for single organ extraction and accurate counting. Especially after the bud stage, cotton leaves overlap severely, and traditional methods, such as K-means clustering [33], Euclidean clustering [34] and conventional region-growing algorithms [35], have difficulty achieving effective segmentation. For this reason, this paper proposes an improved distance-based region-growing algorithm, which can segment organs, such as leaves, stalks and flower buds, of cotton with high accuracy.

The algorithm takes the seed point as the starting point, combines geometric features, such as curvature and normal vector, and introduces the point distance mapping mechanism to effectively improve the accuracy of region subsumption, as in Algorithm 1. Specifically, after processing the seed point, the algorithm computes the Euclidean distances between the unprocessed points and the regions to subsume them into the region with the closest distance, and updates the distance mapping in real time to ensure dynamic accuracy. In addition, the algorithm also has a mechanism for counting the number of region points to eliminate noisy regions with too few points, thus further improving the overall segmentation quality. The method demonstrates excellent adaptability and segmentation accuracy when facing cotton point cloud data with complex structures and serious organ overlap, providing reliable support for subsequent organ-level analysis and counting.
**Algorithm 1:** Improvement of the region growth algorithm**Input: *P*:** cotton seedling individual point cloud 
   ***θ*:** normal vector threshold 
   ***k*:** curvature threshold 
   **N:** maximal number of regions
**Output:** Segmentation result (leaf or stem regions)
1: Initialize U = 0, U′ = U;  // All points are unprocessed
2: Initialize empty regions R[i];  // List to store regions
3: Initialize queue with seed points from *P*
**// Step 1:** Region growing for each seed point based on normal vector and curvature
4: **for** (k = 1 to N) **do**
5:   **for** each point *Pi*
**in** P:
6:    **if**
*Pi* normal_vector < *θ*:
7:     **if**
*Pi* curvature < *k*:
8:      Grow point *Pi* into the nearest region R[min_region_id]
9:      Add *Pi* to region R[min_region_id]
10:     **else**:
11:      Add *Pi* as a new seed point for the next iteration
12:    **else**:
13:     Discard point *Pi*  // Do not grow this point
14:   **end for**
15:   Update regions based on the newly classified points: R = R ∪ R′ 
16:   U = U ∪ U′  // Update the processed points
// **Step 2:** Merge unprocessed points to the nearest region based on Euclidean distance
17: **for** each unprocessed point *Pi*
**in** P do
18:   Find the nearest region R[min_region_id] using Euclidean distance
19:   Add *Pi* to region R[min_region_id]
20: **end for**
// **Step 3:** Return all regions after growing and merging
21: **Return** the segmented regions R[i]

### 2.4. Cotton Phenotype Extraction

**Figure 9:**
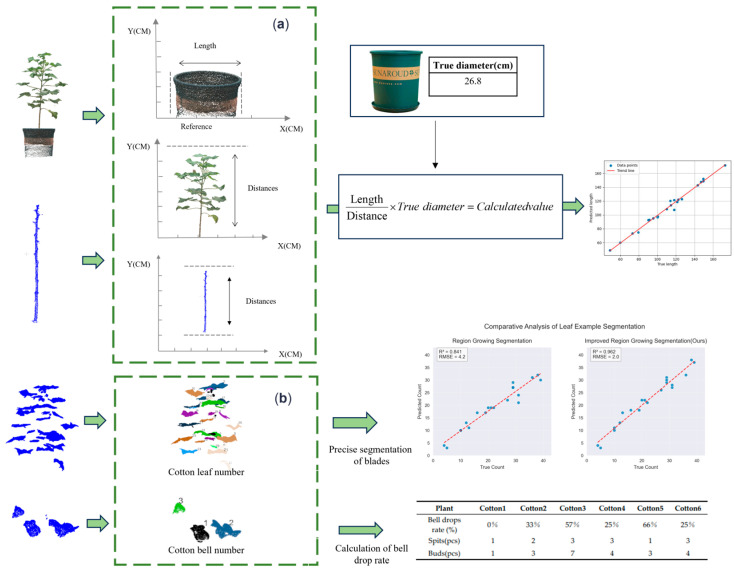
Cotton phenotype extraction process. (**a**) Calculations. (**b**) Precise segmentation of individual organs.

Phenotypic parameters of plants are important indicators for assessing the accuracy of plant reconstruction and segmentation. In this study, two key phenotypic parameters of cotton were extracted—plant height and stem length—and the bell drop rate was used as a growth indicator. Plant height was calculated from the complete plant point cloud data before segmentation using the Euclidean distance algorithm; stem length was calculated based on the main stem point cloud data extracted after segmentation, also using the Euclidean distance algorithm. These phenotypic parameters not only reflect the growth rate and biomass accumulation of plants, but also reveal the adaptability of plants to environmental changes and their physiological responses, as shown in Figure 9.

#### 2.4.1. Calculation of Bell Drop Rate

The bell drop rate is an important indicator of fruit formation, and a high bell drop rate usually signals a reduction in final fruit yield; therefore, accurate calculation of the bell drop rate is crucial for predicting the crop harvest. Based on the improvement of the region-growing algorithm, we are able to accurately segment and count different individuals of the same organ, as shown in Figure 9b. Segmentation of individual organs is a prerequisite to ensure accurate calculations due to gaps between separate individual organs. After segmentation, the bell drop rate was calculated by counting the number of individual organs. In the case of cotton, bud break is calculated by comparing the maximum number of buds (i.e., the maximum number of peaches and buds) of a single cotton plant with the number of mature cottons at the fluffing stage. This method provides a scientific basis for assessing the bell drop rate in cotton, which in turn helps to predict the final yield.

The formula for calculating the bell drop rate is as follows:

(3) $$Sheddingrate=\frac{I-E}{I}\times 100\%$$

In Formula (3), I is the total number of buds counted in a given time period. E is the number of buds that remain alive until flowering or fruiting. Sheddingrate is the bell drop rate.

#### 2.4.2. Calculation of Plant Height and Stem Length

In plant phenotyping studies, plant height and main diameter length are two key parameters for assessing plant growth status and health level. Using the Euclidean distance algorithm, key shape features can be extracted from 3D point cloud or image data of plants, and then plant height and main diameter length can be accurately calculated, as shown in Figure 9a.

Plant height is an important measure of plant growth and is usually determined by measuring the vertical distance between the highest and lowest points of the plant. However, plant growth may show large variations due to changing environmental conditions. The plant height is usually calculated after plant division. First, the point cloud data containing the cotton plant and the planter are rotated so that the planter plane is aligned perpendicular to the *z*-axis and parallel to the horizontal plane. Then, the spatial distance from the base to the top of the plant is calculated as the plant height [36]. The formula for its calculation is as follows:

(4) $$H=Z_{\max}-Z_{\min}$$

In Formula (4), H denotes plant height (plant height), defined as the difference between the highest point of the plant (the Z coordinate of the top point of the plant) and the lowest point (usually the Z coordinate of the ground point) in the point cloud data.

The main path length is calculated from the main stem point cloud data after precise segmentation of individual organs. The Euclidean distance accumulation method is used to gradually calculate the 3D Euclidean distances between neighboring points on the main stem point cloud and accumulate them to obtain the true path length of the main stem [37]. The calculation formula is as follows:

(5) $$L_{\mathrm{stem}}=\sum_{i=1}^{n-1}\sqrt{{\left(X_{i+1}-X_{i}\right)}^{2}+{\left(Y_{i+1}-Y_{i}\right)}^{2}+{\left(Z_{i+1}-Z_{i}\right)}^{2}}$$

In Formula (5), $X_{i}Y_{i}Z_{i}$ denotes the coordinates of the i point in the cloud skeleton of main stem points, and n is the number of main stem points. The formula calculates the sum of the Euclidean distances of all neighboring main stem points, the true path length of the main stem.

## 3. Experimental Results and Analysis

### 3.1. Environment and Setting

Experimental Configuration: To avoid the influence of different experimental conditions on the results of the improved model, all experiments in this study were performed in the same hardware and software environment. The system was equipped with 16 GB of RAM and an NVIDIA GeForce RTX 3060 (NVIDIA Corporation, Santa Clara, CA, USA) graphics card. All experiments were conducted on a Windows 11 (Microsoft, Redmond, WA, USA) Home Edition system, configured with Python 3.9 programming environment, CUDA 11.1.1 architecture, and cuDNN v8.2.0 development library. The deep learning framework PyTorch 1.10.1 was used to build the 3D segmentation networks.

Training Strategy: In the training parameters, the weight decay rate is 0.01, the initial learning rate is 0.001, a total of 200 iterations were performed, and the batch size is set to 2. The sampling points are 2048, and the number of channel dimensions is set to 7 (x, y, z, N_x_, N_y_, N_z_, labels). The k nearest neighbors (required for individual models) in the model are unified to 20. Other parameters are adopted from the original paper recommendations.

### 3.2. Assessment of Indicators

#### 3.2.1. Model Comparison Experiment Evaluation

We used mIoU metrics, mP (mean precision), mR (mean recall), and mF1 (mean F1 score) as the evaluation metrics of cotton organ segmentation for improving the model performance.

mIoU is used to evaluate the performance of the model in the cotton organ segmentation task, measuring the point cloud overlap between the predicted and real regions for each organ category [38], as shown in the following Equation:

(6) $$\mathrm{mIoU}=\frac{1}{N}\sum_{i=1}^{N}\frac{\left|A_{i}\cap B_{i}\right|}{\left|A_{i}\cup B_{i}\right|}$$

$A_{i}$ denotes a set of predicted point clouds for category i, $B_{i}$ denotes a set of real point clouds for category i, and N denotes the total number of organ categories.

mP is a measure of the mean of the model’s precision on each cotton organ class. Precision is the proportion of samples correctly predicted as a positive category over all samples predicted as a positive category, as calculated in the following formulas:

(7) $${\mathrm{Precision}}_{i}=\frac{TP_{i}}{TP_{i}+FP_{i}}$$

(8) $$\mathrm{mP}=\frac{1}{N}\sum_{i=1}^{N}\frac{TP_{i}}{TP_{i}+FP_{i}}$$

$TP_{i}$ denotes the number of true cases in class i, and $FP_{i}$ denotes the number of false positive cases in class i.

mR is a measure of the average of the model’s recall on each cotton organ class. Recall is the proportion of samples correctly predicted to be in the positive category to the true positive category, as calculated in the following formula:

(9) $${\mathrm{Recall}}_{i}=\frac{TP_{i}}{TP_{i}+FN_{i}}$$

(10) $$\mathrm{mR}=\frac{1}{N}\sum_{i=1}^{N}\frac{TP_{i}}{TP_{i}+FN_{i}}$$

$FN_{i}$ denotes the number of false counterexamples in class i.

The mF1 score is the reconciled average of precision mP and Recall. The F1 score takes into account the balance between precision and recall, and larger values are better, as shown in the following formulas:

(11) $$F1_{i}=2\times \frac{{\mathrm{Precision}}_{i}\times {\mathrm{Recall}}_{i}}{{\mathrm{Precision}}_{i}+{\mathrm{Recall}}_{i}}$$

(12) $$\mathrm{mF}1=\frac{1}{N}\sum_{i=1}^{N}\frac{2\times {\mathrm{Precision}}_{i}\times {\mathrm{Recall}}_{i}}{{\mathrm{Precision}}_{i}+{\mathrm{Recall}}_{i}}$$

#### 3.2.2. Phenotype Extraction Assessment

Relative error was used as an evaluation metric for phenotype extraction. The results of phenotypic parameter extraction were evaluated using measured plant phenotypic data, and manually counted plant data.

(13) $$\mathrm{MRE}=\frac{1}{n}\sum_{i=1}^{n}\left|\frac{y_{i}-{\hat{y}}_{i}}{y_{i}}\right|\times 100\%$$

where $y_{i}$ denotes true value, ${\hat{y}}_{i}$ denotes predicted value, and n denotes sample size.

### 3.3. Point Cloud Segmentation Results and Analysis

#### 3.3.1. Network Performance Analysis

**Figure 10:**
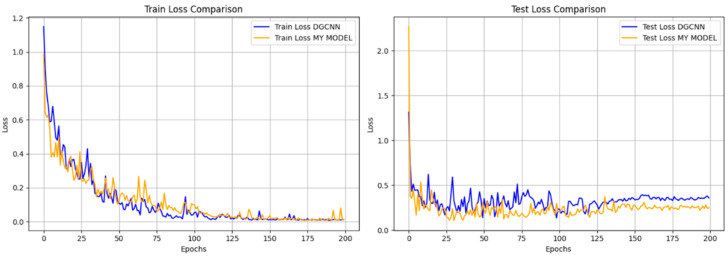
Cotton point cloud organ segmentation loss rate.

In order to deeply explore the training process of the two models, we compare the trend of the loss function during the training process between the improved model and DGCNN, whose loss rate curves are shown in Figure 10. It can be observed that the improved training and testing loss rates fluctuate less, and the difference between the training loss and the testing loss is closer, and the final convergence point shows higher stability. This indicates that the improved model has stronger consistency and generalization ability in full-term organ segmentation in cotton. In contrast, DGCNN has a lower loss function value at the final convergence, but its test loss rate exhibits obvious unstable fluctuations, reflecting a certain bias in its adaptability to the training and test sets. This lack of adaptability may lead to the degradation of segmentation accuracy in practical applications.

Combining the above results, it can be clearly seen that the improved model outperforms DGCNN in the cotton full-phase segmentation task. It not only improves the segmentation accuracy, but also significantly enhances the stability and generalization ability of the model.

#### 3.3.2. Results of Comparative Analysis of Models for Cotton Organ Segmentation

Eight advanced state-of-the-art networks, DGCNN, DeepGCNs [39], Pointnext [40], Pointvector [41], Pix4Point [42], Pointnet, Pointnet++, and Pointnet++ (MSG), were selected to be compared with ResDGCNN in terms of performance and accuracy (Table 1). The experimental environment and parameter settings of the model are as described in Section 2.1 and the other networks are adopted as suggested in their original papers. In order to comprehensively analyze the performance of the network model, we conducted an in-depth experimental analysis of it based on a variety of evaluation metrics, including mIoU, mP, mR and mF1. The experimental results show that the improved model shows absolute advantages in all efficiency metrics. The average accuracy of the improved model is increased by four percentage points on the basis of the original one, and shows a large improvement in mP, mR, and mF1, which implies that the improved network model has good performance in organ segmentation throughout the entire cotton period.

The improved network shows significant improvement in the above metrics (including mIoU, mP, mR, and mF1), which further proves its advantages in point cloud data processing. This performance improvement is mainly due to the excellent performance of the residual module in feature extraction, which enables the network to extract features from different levels and scales, and enhances the accuracy and diversity of feature representations to significantly improve segmentation accuracy.

#### 3.3.3. Results of the Analysis of Cotton Organ Segmentation at Various Periods of Time

**Figure 11:**
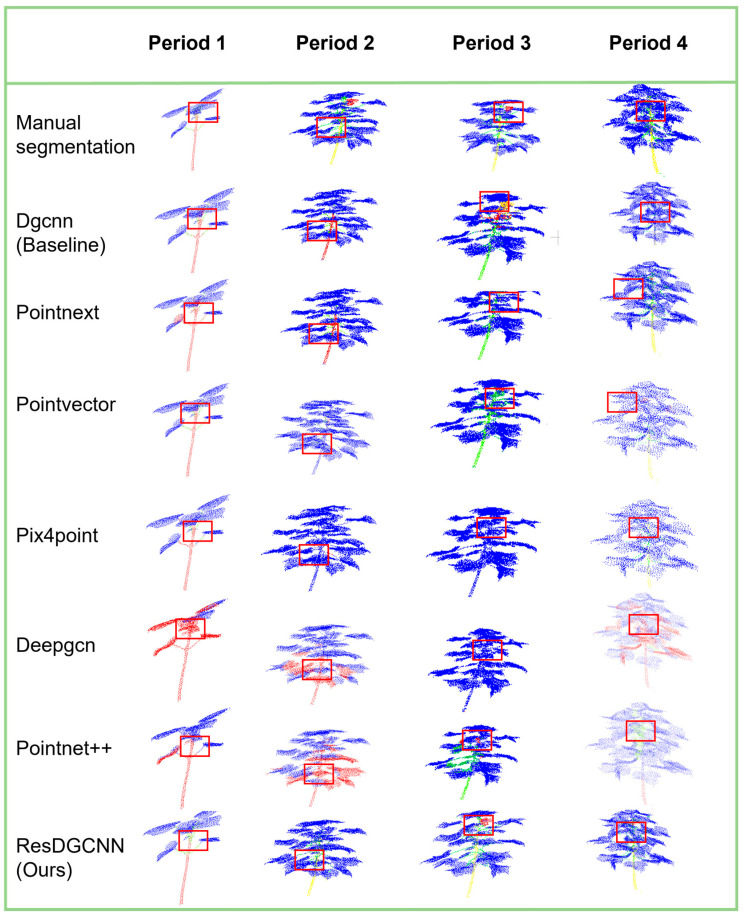
Visualization of organ segmentation model comparison.

In order to more intuitively assess the effectiveness of ResDGCNN’s segmentation of cotton full-period data, we compare the segmentation results of multiple base models in four periods, display the segmentation mIoU of each category in different periods in a table, and generate the corresponding visualization results. From the experimental results, each row in the table shows the performance of different segmentation models when segmenting cotton point clouds at four different cotton growth stages (period 1 to period 4), and the segmentation results are measured by the average mIoU. Taking Figure 11, to visualize the segmentation results of different models in the same period, we use different color schemes to distinguish different periods.

The ResDGCNN model proposed in this study maintains the lead in all periods (Table 2), with the most outstanding performance of 54.18% in the third period, which is a 6.14% improvement over the next best model (DGCNN). The best performance among the traditional methods is PointNet++ (MSG), which shows the advantage of multi-scale feature fusion in period 2 (51.85%), but its performance drops significantly in the more complex period 4 (39.88%), which shows that it is less effective in feature extraction on small sample data. The overall performance of all models showed significant cycle dependence. Period 1 (three components) had the best overall performance (68.9% on average), while most of the models declined in performance as the number of components increased. This reflects the effect of the small percentage of data in cotton for special component organs on model training.

In period 1, the core of model differentiation is reflected in the segmentation accuracy of the stem and main stem, where the distinction between stem and leaf is especially critical. Experimental results show that the ResDGCNN model exhibits better performance in the stem–leaf segmentation task compared to PointNext and DGCNN. The segmentation of the main stem becomes the most challenging task from period 2 to period 4. It is worth noting that the DGCNN model incorrectly recognizes buds (red labeling) as flowers (yellow labeling), while the ResDGCNN model achieves accurate recognition and segmentation.

Currently, the experiment compares the performance of multiple point cloud segmentation models in four growth periods (periods 1–4) of cotton, and the results show that the improved DGCNN model achieves the leading position in all the phases, especially in period 3 and period 4, where the organ structure is complex and the number of categories increases, with accuracy reaching 54.18% and 60.96%, which is significantly higher than other models. It also achieves a high accuracy of 77.77% in period 1, demonstrating excellent early recognition capability. Currently, ResDGCNN tops all the models in terms of average accuracy, shows great stability and generalization ability, and is especially suitable for dealing with the task of plant point clouds with complex structures and few samples.

#### 3.3.4. Cotton Organ Segmentation Results of Different Organ Analysis

To further validate the network performance in the task of point cloud segmentation of individual plant organs, we compared the performance of nine typical point cloud segmentation networks over four different time periods (periods 1–4) against a dataset containing five categories labeled with different periods: leaf, stem, mainstem, flower and peach. All models were evaluated under the same training strategy and testing conditions, and the table shows the segmentation mIoU (%) for each category at different time periods (Table 3).

Among the five categories of plant organs, the leaf category has the most obvious structure and is the most accurately recognized part of each model at different growth stages. PointVector performed the best in the leaf segmentation task, reaching a peak accuracy of 99.79% in period 1. In contrast, the stem and mainstem, which are similar in structure and less discriminative, are the key components that affect the overall segmentation task performance, placing higher demands on the model’s performance. In the segmentation task of these two stem categories, PointNet++ (MSG) achieved the best results, showing its stronger structural recognition ability. Flower and peach, as the organ categories with the least number of samples, had relatively lower overall segmentation accuracy. However, DGCNN and its improved version with the introduction of the attention mechanism show greater advantages in small-sample organ segmentation, generally outperforming traditional methods.

In the task of multi-organ segmentation in four growth cycles, the models performed differently, which significantly reflected the characteristics of the models in dealing with complex plant structures. Among them, ResDGCNN shows a strong comprehensive ability in multiple stages, especially in the segmentation task of different organs with high generalization performance. In terms of segmentation accuracy for some specific organs (leaf and stem), ResDGCNN does not always achieve the highest scores, but its average performance is more balanced across all organ classes, and the overall segmentation results are better than those of other models, thanks to the structural improvements brought by its introduced residual module. The residual module can effectively alleviate the problem of gradient vanishing during the training process of the deep network, and at the same time, it enhances the stability and robustness of feature extraction, so that the model can still maintain high accuracy and stability in the face of the actual situation of large variations in organ morphology and sample imbalance.

Currently, by comparing and analyzing nine point cloud segmentation models over four growth cycles of five types of plant organs, the results show that, although ResDGCNN is not the best performer on some organs (leaf or stem), with the introduced residual module, it significantly improves the stability and robustness of the model in feature extraction, and shows excellent generalization ability in the segmentation task with multiple categories, small samples and complex structure, which makes it an effective solution in the joint segmentation of multiple organs.

#### 3.3.5. Results of Precise Segmentation of Individual Organs in Cotton

**Figure 12:**
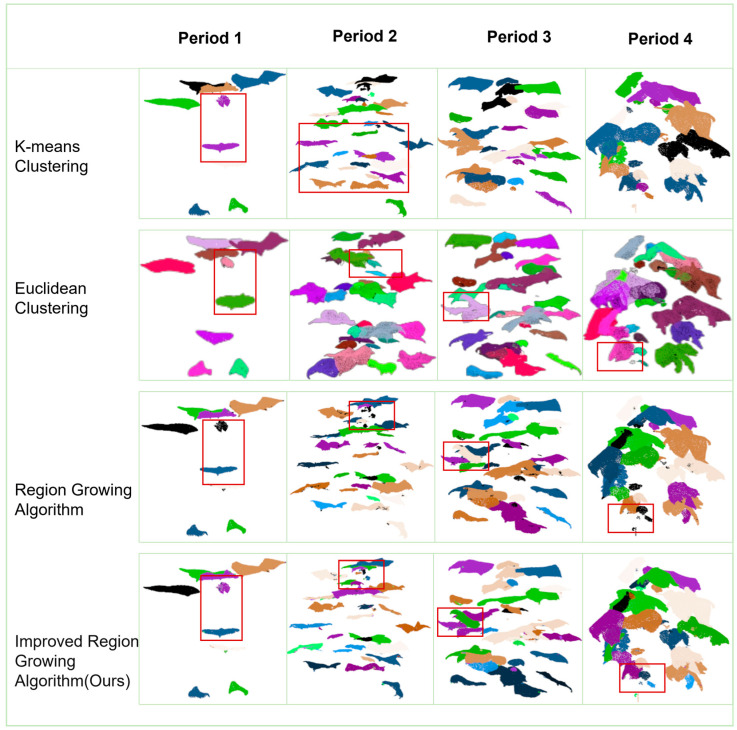
Comparison of cotton example segmentation visualization.

**Figure 13:**
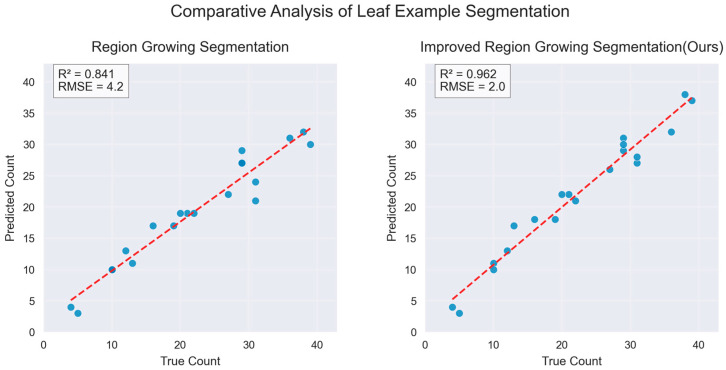
Regression analysis of cotton leaf precise segmentation of individual organs.

On the basis of point cloud part segmentation, the best segmentation effect is achieved by using an improved region-growing algorithm for accurate segmentation of segmented cotton organs. The segmentation compares the effect of K-means clustering, Euclidean clustering, and the region-growing algorithm in segmenting cotton at different periods, and the segmentation results are shown in Figure 12. Twenty cotton leaf samples were selected, and the number of clusters was calculated using the region-growing algorithm and the improved region-growing algorithm and compared with the real number; the comparison results are shown in Figure 13.

The point cloud files segmented using the K-means clustering method are missing foliage information for leaf segmentation at the seedling stage and do not achieve accurate segmentation in other periods. Both methods, using Euclidean clustering and region growing, segmented most of the leaves proficiently on cotton leaves at the full stage. However, the small, newly grown cotton leaves did not accomplish segmentation in the Euclidean clustering method. The European clustering method used direct removal for a small range of small aggregates, which significantly affected the segmentation of small leaves. The region-growing algorithm was found to be the most effective among the three segmentation methods in the comparative study by effectively segmenting most of the leaves at the full stage, while preserving the point cloud information of the small leaves. However, the region-growing algorithm was found to be ineffective in segmenting small leaves with a short growth cycle in post-seedling (bud and fluffing) cotton leaves and only retained information. The improved region-growing algorithm effectively performed the segmentation of cotton leaves at the full stage, and achieved good segmentation results for leaves of different sizes.

The number of cotton leaves in different periods varies greatly, and the segmentation difficulty also varies. The number of seedlings and seedling cotton leaves is about 2–20 pieces; in this cycle, the number of cotton leaves is small, leaf spacing is large, and point cloud segmentation difficulty is small. In the bud stage, there are mostly 20–30 cotton leaves; in this cycle, cotton segmentation is difficult, and there are more noise points. Segmentation is performed using an improved region-growing algorithm. The core of the region-growing algorithm is to sort the curvature values and take the point with the smallest curvature as the initial seed point; in the cotton point cloud segmentation of the individual organ data, the curvature is one of the attributes of the point cloud data, and the simple region-growing algorithm does not make use of the other attributes of the point cloud, such as the color, the distance and other attributes. Based on this idea, the experiments began to try to add further segmentation of color to the distance and other information; it was found that the improved region-growing algorithm with the addition of color was not achieved, which is the reason for the strong color similarity of point clouds in similar organs. While distance is the core of several clustering algorithms, using the constraint of distance removes noisy points while retaining small blades. In the comparison between the improved segmentation results and the original region-growing algorithm, it can be seen that before the improvement, it is black (unsegmented), and after the improvement, it is multi-colored (segmented), as shown in Figure 12. Quantitative segmentation results are shown in Figure 13.

In general, cotton organs are diverse and leaf segmentation is the most representative method for accurate segmentation of cotton. In the seedling stage and the emergence stage of cotton, the segmentation goal can be achieved by using non-improved algorithms (Euclidean clustering and region-growing algorithms) for cotton leaves with only a few leaves. The improved region-growing algorithm has better segmentation performance for 20–30 leaves at the bud stage and the floret stage of cotton. The judgment mechanism based on point distance mapping in the improved algorithm enhances the completeness of individual leaf segmentation and retains more point cloud information of the leaves. The statistical mechanism for the number of points in the region increases the accuracy of the segmentation of small leaves based on the removal of noisy points. The improved region-growing algorithm shows remarkable robustness and accuracy for full-term complex cotton single leaves.

### 3.4. Results of Phenotypic Parameter Extraction

For the segmented organs of a single cotton plant, picked from 300 cotton point cloud data at the bud stage and spitting stage, the bell drop rate of six cotton plants was calculated using the improved region-growing algorithm, with the results shown in the table. In the table, the first row is the bell drop rate, the second row is the number of cotton buds, and the third row is the number of flower buds. From this, we can see that the bell drop rate of plant 1 is the smallest at 0%, because plant 1 has the fewest buds, at only one. Plant 5 has the largest percentage of de-budding at 66%. The overall bud release rate of cotton from plant 2 to plant 6 tends to become smaller, which corresponds to the distribution of cotton in Figure 1b. Cotton bud-breaking and de-breaking have a significant relationship with pollination at the flowering stage, and under natural conditions, cotton is mostly wind pollinated. In the greenhouse, the number of cotton plants is small, pollination during flowering is difficult, and is greatly affected by wind speed; the number of cotton buds generally corresponds to the direction of the wind, and the trend is increasing. Due to planting reasons, the growth of Plant 5 in the seedling stage is affected, a large number of leaves fall off, the flowering stage is later than the other plants, pollination is difficult, and it has the highest bell drop rate (Table 4).

**Figure 14:**
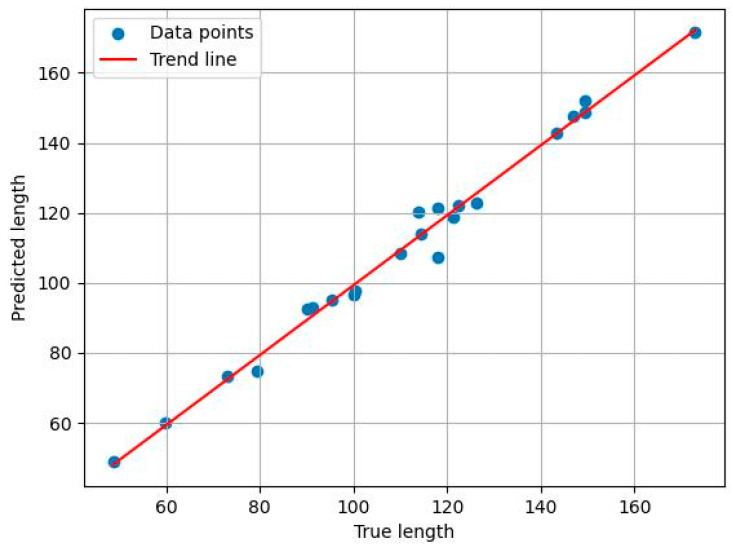
Parameter extraction correlation.

Twenty cotton seedling indexes were selected, the parameters were extracted from the cotton main stem, and the correlation between the phenotypic parameter estimates and manual measurements was assessed as shown in Figure 14. The average relative error between the main stem calculations and manual measurements was 0.973, and there was a high correlation between the estimates and manual measurements.

Currently, the bell drop rate was calculated using an improved area growth algorithm, and the results were consistent with the growth characteristics of each plant. The average relative error between the calculated and manual measurements was 0.973 for the cotton main stem length measured using the reference comparison, and there was a high correlation between the estimated values and the manual measurements.

## 4. Discussion

In this section, we discuss three parts: the effect of external perturbations on the 3D reconstruction of plants; the effect of the number of training points on the effectiveness of point cloud segmentation; and the optimization of region-growing segmentation guided by multi-scale features.

**Figure 15:**
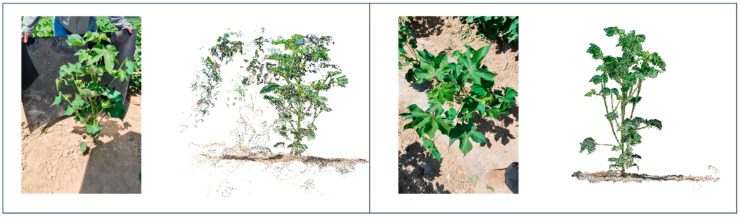
Effects of reconstruction of the test field.

(1) For the effect of external perturbations on the 3D reconstruction of plants, this paper adopts the 3D reconstruction method based on the neural vector field to process the video data captured by cell phone. The method has the advantages of low cost [43] and high reconstruction accuracy [44], and the resulting point cloud data can better meet the subsequent experimental needs of organ segmentation and phenotypic parameter extraction. In order to better observe the morphological characteristics of cotton at various growth stages, most of the data acquisition work was carried out in a greenhouse environment. However, some cotton plants were also moved outdoors at specific time periods for photographing. Comparative analysis showed that the data collected outside the greenhouse did not differ much from the data inside the greenhouse in terms of reconstruction effect under small external stimulus conditions, and all of them were able to capture the details of the plants better. Based on this finding, a further attempt was made to perform video acquisition and 3D reconstruction in the experimental field. The results showed that compared with the acquisition method under greenhouse conditions, the video captured directly in the natural environment had a poorer reconstruction effect with significantly more background noise, and the reconstruction effect of the data captured in the experimental field is shown in Figure 15.

Under the same outdoor conditions, although the reconstruction of cotton data collected outside the greenhouse was better, the cotton reconstruction results in the experimental field showed significantly more noise points, a phenomenon that caught my attention. After comparatively analyzing the experimental environment, I found that external perturbation factors, such as wind, had a significant effect on the plant reconstruction process. As typical flexible organisms, plants are highly dynamic in their morphology. In natural environments, plants not only produce responsive deformation to external stimuli, such as wind, but also exhibit significant circadian rhythms and seasonal growth characteristics. These dynamic changes make 3D reconstruction in field environments more challenging, especially under conditions of high wind speed, which can easily lead to morphological inconsistencies between image frames, thus destroying the training stability and reconstruction continuity of the neural rendering network.

(2) The impact of the number of training points on the effect of point cloud segmentation. In the prediction of the model segmentation results, this paper found the number of samples has a great impact on the model segmentation accuracy (Table 5). In periods 1–4 of cotton training using DGCNN training, for example, the model trained using 2048 points shows poorer results in the prediction of other points. In further research, it may be considered to increase the number of trained plant point clouds, thereby achieving better segmentation results for plants [45].
(3) Multiscale feature-guided optimization of regional growth segmentation aims at the problem that different cotton organs have local overlapping and fuzzy boundaries in space, which makes it difficult to segment them accurately. In this paper, based on the seven-dimensional feature information (x, y, z, N_x_, N_y_, N_z_, labels) contained in the point cloud dataset, we combine the point cloud coordinate features and normal vector features for the region growth determination and expansion. Two optimization strategies, point distance mapping and curvature normal vector, are introduced to design and implement an improved region-growing algorithm. The algorithm can realize fine-grained precise segmentation of multiple individual organs (including leaves, stalks, flower buds, etc.) of cotton, so as to distinguish organ boundaries more effectively and inhibit the erroneous fusion of overlapping regions.

The improvement strategies proposed in this paper are mainly for 3D point cloud data with normal vector information. When applied to other point cloud precise segmentation of individual organs tasks, if the data contain multi-dimensional perceptual information, such as color [46], texture [47], curvature [48], and semantic information [49], strategies, such as color gradient and multi-scale structural features, can be further introduced to optimize the region-growing algorithm in a specific way, in order to better fit the specific experimental needs and segmentation goals.

## 5. Conclusions

This study focuses on the acquisition of 3D phenotypic data of cotton under greenhouse growth conditions and proposes a high-precision point cloud segmentation method that integrates ResDGCNN with an improved region-growing algorithm. A comprehensive 3D point cloud dataset of cotton covering the entire growth period was also constructed. The proposed ResDGCNN model enhances the segmentation performance of organs at various developmental stages by combining residual learning with dynamic graph convolution mechanisms. To address the challenge of complex overlaps between organs, the study introduces point distance mapping and curvature-based normal vector information into a modified region-growing algorithm, enabling fine segmentation of organs, such as leaves, stems, and buds. The phenotype extraction framework built upon the segmentation results demonstrates high accuracy and stability, effectively supporting dynamic growth analysis and trait evaluation of cotton. The proposed method shows significant advantages in cotton phenotypic analysis and growth monitoring, providing reliable technical support for precision agriculture and intelligent breeding. Future research may focus on scaling up the method for larger field trials and optimizing the algorithm to accommodate a wider range of crops and more complex environments.

## Figures and Tables

**Table 1:** Model performance analysis.

Model	mIoU (%)	mP (%)	mR (%)	mF1 (%)
DGCNN (Baseline)	62.69	67.43	71.52	69.42
DeepGCNs	52.78	59.63	66.68	62.97
Pointnext	61.90	66.98	72.35	69.57
Pointvector	60.02	66.21	73.15	69.52
Pix4Point	53.79	60.74	68.94	64.59
Pointnet	53.49	67.50	71.53	69.46
Pointnet++	61.43	67.81	72.24	69.96
Pointnet++ (MSG)	63.54	68.51	73.20	70.78
ResDGCNN (Ours)	**67.55**	**71.76**	**77.37**	**74.46**

**Table 2:** Analysis of different periods of the model.

Model	mIoU (%)			
	Period 1	Period 2	Period 3	Period 4
DGCNN (Baseline)	69.39	46.01	48.04	61.34
DeepGCNs	57.50	46.31	17.30	57.38
Pointnext	76.44	43.59	42.13	50.66
Pointvector	67.87	47.17	44.88	53.40
Pix4Point	64.24	38.63	17.30	52.46
Pointnet	62.80	43.41	32.25	48.23
Pointnet++	73.77	43.58	35.26	55.16
Pointnet++ (MSG)	72.22	51.85	39.88	60.96
ResDGCNN (Ours)	**77.77**	**53.33**	**54.18**	**66.45**

**Table 3:** Analysis of different organs at different periods of the model.

Period	Model	mIoU (%)				
		Leaf	Stem	Mainstem	Flower	Peach
Period 1	DGCNN (Baseline)	93.60	45.35	69.24	-	-
	DeepGCNs	93.47	26.84	52.21	-	-
	Pointnext	97.44	52.53	79.37	-	-
	Pointvector	**99.79**	45.09	64.55	-	-
	Pix4Point	95.23	25.30	72.20	-	-
	Pointnet	97.60	43.83	66.22	-	-
	Pointnet++	97.93	54.75	76.65	-	-
	Pointnet++ (MSG)	96.84	47.51	**88.90**	-	-
	ResDGCNN (Ours)	98.42	**71.63**	87.89	-	-
Period 2	DGCNN (Baseline)	91.24	37.06	51.92	3.82	-
	DeepGCNs	93.80	40.24	51.22	0.00	-
	Pointnext	92.37	23.36	**58.65**	0.00	-
	Pointvector	94.91	**48.91**	44.88	0.00	-
	Pix4Point	91.36	17.09	46.09	0.00	-
	Pointnet	94.62	19.1	46.4	0.23	-
	Pointnet++	**96.48**	24.32	43.62	9.75	-
	Pointnet++ (MSG)	96.34	18.33	41.92	26.65	-
	ResDGCNN (Ours)	92.51	40.17	54.02	**31.11**	-
Period 3	DGCNN (Baseline)	92.74	41.96	61.10	23.06	21.37
	DeepGCNs	86.55	0.00	0.00	0.00	0.00
	Pointnext	94.52	43.95	68.90	0.00	3.28
	Pointvector	93.77	43.32	66.26	0.00	0.00
	Pix4Point	86.55	0.00	0.00	0.00	0.00
	Pointnet	93.34	24.62	7.72	3.41	3.33
	Pointnet++	95.24	34.38	60.37	0.00	5.61
	Pointnet++ (MSG)	94.00	33.95	78.42	**56.55**	9.10
	ResDGCNN (Ours)	**94.53**	**46.62**	**83.26**	28.88	**38.69**
Period 4	DGCNN (Baseline)	91.85	**43.41**	63.03	-	47.10
	DeepGCNs	96.22,	38.62	64.37	-	30.34
	Pointnext	93.66	18.58	61.34	-	29.09
	Pointvector	93.81	24.71	55.37	-	39.74
	Pix4Point	94.80	35.56	**69.67**	-	9.80
	Pointnet	92.72	23.12	55.33	-	1.92
	Pointnet++	94.55	32.22	64.36	-	6.31
	Pointnet++ (MSG)	92.12	40.13	60.71	-	**50.90**
	ResDGCNN (Ours)	**96.80**	43.21	69.43	-	38.38

**Table 4:** Bell drop rate of different plants.

Plant	Cotton1	Cotton2	Cotton3	Cotton4	Cotton5	Cotton6
Bell drops rate (%)	0%	33%	57%	25%	66%	25%
Spits (pcs)	1	2	3	3	1	3
Buds (pcs)	1	3	7	4	3	4

**Table 5:** Effect of sampling points on test results.

Point Number	mIoU (%)		
	Leaf	Stem	Mainstem
1024	94.17	47.20	83.3
2048	97.30	**58.33**	75.52
3072	**97.99**	45.75	**79.41**
4096	97.72	42.91	77.93
5120	97.52	38.15	76.16
10240	94.45	4.50	72.60
20480	92.99	1.16	71.73

## Data Availability

The data presented in this study are available on request from the corresponding author. The data are not publicly available due to privacy.
